# Effects of Combined Abiotic Stresses Related to Climate Change on Root Growth in Crops

**DOI:** 10.3389/fpls.2022.918537

**Published:** 2022-07-01

**Authors:** Maria Sánchez-Bermúdez, Juan C. del Pozo, Mónica Pernas

**Affiliations:** Centro de Biotecnología y Genómica de Plantas (CBGP), Universidad Politécnica de Madrid and Instituto de Investigación y Tecnología Agraria y Alimentaria-Consejo Superior de Investigaciones Científicas (UPM-INIA/CSIC), Campus de Montegancedo, Madrid, Spain

**Keywords:** climate change, root traits, crop yield, crop adaptation, abiotic stresses, combined stresses

## Abstract

Climate change is a major threat to crop productivity that negatively affects food security worldwide. Increase in global temperatures are usually accompanied by drought, flooding and changes in soil nutrients composition that dramatically reduced crop yields. Against the backdrop of climate change, human population increase and subsequent rise in food demand, finding new solutions for crop adaptation to environmental stresses is essential. The effects of single abiotic stress on crops have been widely studied, but in the field abiotic stresses tend to occur in combination rather than individually. Physiological, metabolic and molecular responses of crops to combined abiotic stresses seem to be significantly different to individual stresses. Although in recent years an increasing number of studies have addressed the effects of abiotic stress combinations, the information related to the root system response is still scarce. Roots are the underground organs that directly contact with the soil and sense many of these abiotic stresses. Understanding the effects of abiotic stress combinations in the root system would help to find new breeding tools to develop more resilient crops. This review will summarize the current knowledge regarding the effects of combined abiotic stress in the root system in crops. First, we will provide a general overview of root responses to particular abiotic stresses. Then, we will describe how these root responses are integrated when crops are challenged to the combination of different abiotic stress. We will focus on the main changes on root system architecture (RSA) and physiology influencing crop productivity and yield and convey the latest information on the key molecular, hormonal and genetic regulatory pathways underlying root responses to these combinatorial stresses. Finally, we will discuss possible directions for future research and the main challenges needed to be tackled to translate this knowledge into useful tools to enhance crop tolerance.

## Introduction

Climate change is having a harsh impact on natural ecosystems and agricultural production (Watts et al., 2021). Human activities have increased CO_2_ accumulation and other greenhouse gases in the atmosphere to dangerous levels during the last century leading to global warming and other climatic consequences (Chaudhry and Sidhu, 2021). Increase in the frequency of extreme weather events such as heat-waves, drought periods, intense precipitations, flooding, and changes in the freezing patterns are having a strong impact on agricultural production and food security worldwide (Dempewolf et al., 2014). A major challenge for agriculture is to feed an increasing human population in the backdrop of production losses provoked by climate change and at the same time mitigate its effects on the environment. Finding sustainable solutions for crop adaptation to changing climatic conditions and to enhance crop production is key to guarantee food security around the globe (St. Clair and Lynch, 2010). Abiotic stress events are more frequent causing extensive yield losses in many crops around the world (Mittler, 2006; Chaudhry and Sidhu, 2021). Wheat, rice, maize, and soybean, which supply two-thirds of the human food worldwide are expected to suffer yield losses of 6.0, 3.2, 7.4, and 3.1% respectively, for each degree-Celsius increase in the global mean temperature (Zhao et al., 2017). Effects linked to high temperature include changes in shoot and root growth, biomass production, flowering time, and seed production that in turn affect crop yield (Li et al., 2015). Drought is one of the major causes hampering agricultural production over the last 50 years. Thus, a severe reduction in global crop yield due to water deprivation is expected in the near future. Yield-related effects of drought in plants are alterations in different phenological stages, root architecture, seed production, crop biomass, harvest index and germination rates (Pervez et al., 2009; Dietz et al., 2021). Major advances have been made to gain knowledge on the effects of abiotic stresses in crop physiology and development. But we still need to fully understand the complex regulatory mechanisms underlying plant response to be able to enhance crop tolerance (Zhang et al., 2022).

In the last few decades, plant researchers have mainly focused on studying the effects of individual abiotic stresses on plants. However, field conditions are usually much more complex and abiotic stresses tend to occur in combination rather than individually (Suzuki et al., 2014). Concurrent abiotic stresses are often more damaging to crop productivity, especially when they occur at particular stages of plant growth related to crop yield, such as flowering time or seed production. They also have a strong effect on the early stages of plant development when roots are crucial for plant anchorage and settling. Crop productivity is differently affected by combinations of stresses than by individual stresses. The global yield of wheat and maize has been reduced due to the effects of high temperature, and this reduction has been intensified in areas also affected by drought (Matiu et al., 2017). Plants seem to perceive and integrate combined stress signals differently according to the nature of the combined interaction (Pandey et al., 2015). Thus, the physiological, metabolic and molecular response of plants to a particular combination of stresses can be different than individual stresses (Mittler, 2006). Even more, some stress combinations might trigger different signaling pathways in the plant interfering or even producing antagonistic responses compare to their response to individual stresses (Atkinson and Urwin, 2012). For example, during heat stress, plants open their stomata to reduce leaf temperature. But when heat stress is combined with drought, the stomata remain closed and leaf temperature cannot be compensated (Reynolds-Henne et al., 2010). Conversely, the production of reactive oxygen species (ROS) under combined drought and O_3_ stress is lower than under drought stress alone (Iyer et al., 2013). Although some advances have been made, there is still a large gap in our knowledge of the effect of simultaneous stress exposure in crops.

Roots play an essential role in the survival and development of the plant. Roots perform essential functions for the crops such as anchoring them to the soil, providing mechanical support, assimilating water and nutrients and establishing beneficial relationships with the microbiota (Shekhar et al., 2019). Since roots are in direct contact with the soil, they can be considered as the primary sensors of many abiotic stresses, such as drought, salinity or waterlogging (Wells and Eissenstat, 2002; Davies and Bacon, 2003). Roots are also able to trigger specific signaling pathways to adjust their developmental program to survive those stresses (Khan et al., 2016). Abiotic stresses can cause alterations in root morphology and physiology that change their functionality and affect the aerial parts of the plant compromising crop productivity (Ghosh and Xu, 2014). Despite this crucial role of roots, the effects of abiotic stresses on the RSA have been much less studied than on the aerial parts (Koevoets et al., 2016; Calleja-Cabrera et al., 2020). One of the main underlying reasons are the limitations and difficulties associated to the study of the root system in the field (Franco et al., 2015; Atkinson et al., 2019). This limitation has led to the analysis of the root system in controlled conditions, but the environmental setting in enclosed ecosystems tend to underrepresent the climatic conditions that occur in the field (Voss-Fels et al., 2018; Qiao et al., 2019). There has been little research about the effects of abiotic stresses on plant roots and this knowledge is even more limited when it concerns the effects of abiotic stress combination. Consequently, plant breeding has focused primarily on aboveground traits. A change of focus towards the study of the effects of abiotic stress combination in root traits might provide new knowledge and tools for breeding programs focused on improving crop adaptation (Villordon et al., 2014).

This review seeks to summarize the current information on the physiological, hormonal, and molecular effects of abiotic stress combination on the root system. We will provide an overview of the root responses that are shared under different abiotic stresses, and a detailed information about the root responses that are unique to each stress combination. We will describe the main mechanisms regulating crop tolerance and adaptation, focusing on the abiotic stress combinations that are more likely to occur under the scope of climate change. Finally, we will consider future alternatives and venues for research on the root response to combined stresses that could be relevant to tackle the challenge of improving crop tolerance to climate change.

## Common Effects of Abiotic Stresses on the Crop Root System

Abiotic stresses have a big impact on root development and functionality, altering the uptake of water and nutrients as well as the root interactions with microorganisms in the rhizosphere (Koevoets et al., 2016; Choi et al., 2021). Under normal conditions, roots absorb water and nutrients from the soil, providing the cells with essential solutes and water to maintain the appropriate cellular homeostasis and nutrient balance. Under abiotic stresses, roots undergo a series of structural and functional alterations to mitigate the adverse effects on the plant growth caused by the alteration of this balance (Ghosh and Xu, 2014). These changes in RSA and functionality also affect the aerial parts of the plant, altering important physiological and developmental processes like stomatal conductance, photosynthesis or carbon allocation (Khan et al., 2016). All these adjustments in the above and below ground must be coordinated to provide a global plant response and enable stress adaptation.

In response to different abiotic stresses, roots display shared or unique physiological and morphological responses. Examples of shared changes on RSA and physiology are alterations in root length and depth, changes in lateral root formation and elongation as well as alterations in root hair development and carbon and nutrient root-shoot allocation (DoVale and Fritsche-Neto, 2015). Drought stress alters RSA decreasing root biomass and subsequent increase on the root/shoot ratio, and the amount of root carbohydrate and nitrogen content in several crops (Pedroso et al., 2014; Xu et al., 2015; Yıldırım et al., 2018). Heat stress also alters root biomass and growth mainly by modifying the number and elongation of lateral roots (Huang et al., 2012). Phosphate starvation produces an increase in lateral root formation and elongation (Waidmann et al., 2020) and the stress caused by root illumination can also lead to changes in RSA and the shoot:root ratio (Miralles et al., 2011; Silva-Navas et al., 2015).

Some metabolic changes are common to several abiotic stresses. One example is the accumulation of osmoprotectants to avoid intracellular water loss caused by several stresses including drought and salinity. Plants accumulate these metabolites inside the cell to reduce the water potential and promote osmotic adjustment that alleviates cellular damage. This accumulation of osmoprotectants, including amino acids like proline, sugars, oxalate and malate acids or other compounds such as glycine betaine and polyamines, has been observed in different crops exposed to salinity, drought, flooding, temperature changes and heavy metals (Hossain et al., 2022). ROS are natural compounds produce during the cellular metabolism that can act as cellular secondary messengers (Jaspers and Kangasjarvi, 2010). Under the vast majority of abiotic stresses, including drought, salinity, temperature stresses, nutrient deficiency, O_3_ stress or hypoxia, ROS accumulate at much higher levels, causing damage to DNA, carbohydrates and proteins (Moller et al., 2007; Foyer and Noctor, 2009). This accumulation of ROS by abiotic stress has been described in many crops such as wheat, rice, soybean, and tomato (Zhao et al., 2001; Kamal and Komatsu, 2015; Khan et al., 2019; Kushwaha et al., 2019). On the other hand, plant cells contain antioxidative compounds and enzymes to prevent this excessive ROS accumulation and balance the oxidative stress response. Some of those compounds that includes carotenoids, like glutathione and ascorbate, and enzymes such as catalase, superoxide dismutase (SOD) and ascorbate peroxidase (APX) are also induced by abiotic stresses (Desikan et al., 2004). Another common response to abiotic stresses is the increase in cytosolic Ca^2+^ levels due to the activation of Ca^2+^ channels produced in turn by increased levels of ROS and H_2_O_2_ (Ranty et al., 2016). Although this is a crucial cellular response, the calcium channels involved in abiotic stress-responses in roots are poorly known (Wilkins et al., 2016). These changes in Ca^2+^ influx are the signal to trigger several cascades of phosphorylation and dephosphorylation by Ca^2+^-dependent protein kinases. Then, these cascades will transmit the stress signal to the downstream targets of these kinases that includes specific families of transcription factors (TFs) regulating the global stress plant response (Kumar et al., 2020).

Several plant hormones like abscisic acid (ABA), jasmonic acid (JA), ethylene (ET), or salicylic acid (SA) have shown to mediate abiotic stress responses in roots. Thus, ABA acts as intermediary molecule in the root response to stresses such as drought, heat and salinity in wheat, rice, quinoa, cucumber and rice (Talanova et al., 2003; Trapeznikov et al., 2003; Jacobsen et al., 2009; Ding et al., 2016). Moreover, genes involved in the ABA biosynthetic pathway are activated and mediate root growth under difference abiotic stresses (Tuteja, 2007). JA also participates in the crop root response to many abiotic stresses like salinity, drought and thermal stress (de Ollas et al., 2013; Habibi et al., 2019; Ali et al., 2020). Several root responses like salinity in tomato; heat in artichoke; or cold in tomato are mediated by ET (Karni et al., 2010; Klay et al., 2014; Shinohara et al., 2017). SA is also induced in response to drought in barley (Bandurska, 2005). Additionally, root irrigation with exogenous SA induces tolerance to salinity, chilling and heavy metal stress in crops like barley, rice and tomato (Metwally et al., 2003; Stevens et al., 2006; Guo et al., 2009). Other hormones associated with the response to abiotic stresses are cytokinins, brassinosteroids, auxins and gibberellins (Peleg and Blumwald, 2011). For example, barley lines overexpressing a gene encoding for the cytokinin dehydrogenase in roots show more tolerance to drought stress (Pospíšilová et al., 2016) and exogenous application of auxin to salt-stressed strawberry seedlings protects root growth (Zhang et al., 2021).

Abiotic stresses induce global transcriptomic reprogramming to adjust plant growth to the new environmental situation. Several molecular studies have compared the transcriptome of plants exposed to different abiotic stresses and have found overlapping transcriptional patterns in crops like soybean, rice, and barley (Ozturk et al., 2002; Yun et al., 2012; Kidokoro et al., 2015). Plant responses to abiotic stresses also comprise different regulatory gene networks involving specific set of TFs. Members of the MYB family are activated by stresses like drought, UV-light and cold in several crops (Vannini et al., 2007; Schenke et al., 2011; Seo et al., 2011). The NAC family of TFs also plays a role in the response to drought, salinity, cold and dehydration in wheat and rice (Fujita et al., 2004; Nakashima et al., 2007; Seo et al., 2010; Xia et al., 2010). The ERF family of TFs participate in the response to drought, salinity and cold in rice, soybean, and tobacco (Guo et al., 2004; Cao et al., 2006; Zhang G. et al., 2009). The WRKY family is involved in the response to drought and heat rice (Qiu and Yu, 2009; Peng et al., 2011) and the DREB family has been related to drought, salinity and cold stresses in crops like wheat, barley, rice, and soybean (Shen et al., 2003; Li et al., 2005; Wang et al., 2008; Xu et al., 2009). Finally, heat shock factors (HSFs) are crucial TFs involved in the response to numerous abiotic stresses. HSFs regulate the expression of several heat shock proteins (HSPs) that function as molecular chaperons to protect and stabilize essential proteins, preventing their denaturation during stress (Atkinson and Urwin, 2012). Specific HSPs are activated under different abiotic stress conditions and their functional diversity allows plants to respond to a wide range of stresses (Rizhsky et al., 2004). They also mediate the reduction of ROS levels and activate downstream pathways to protect the plant from an excessive oxidative stress (Miller and Mittler, 2006). The role of HSPs as regulators of abiotic stress responses has been reported in several crops like wheat, tomato, or soybean (Mishra et al., 2002; Zhu et al., 2006; Xue et al., 2014).

**FIGURE 1:**
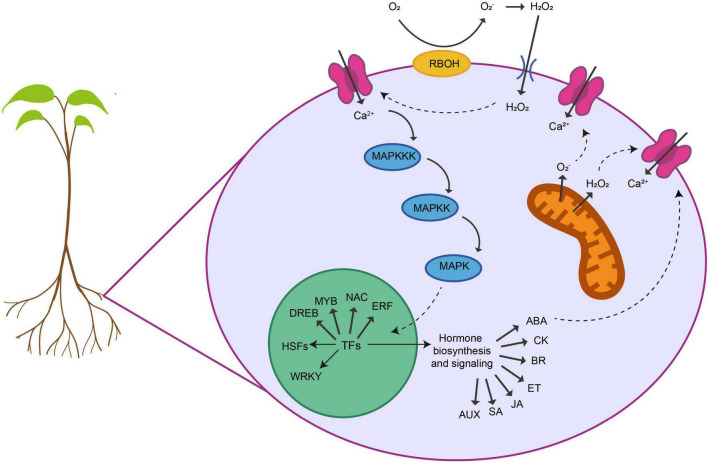
Shared responses of plant roots to abiotic stresses. Roots display different cellular and molecular responses to alleviate the negative effects in their development and functionality provoked by abiotic stresses. Several key sensing, signaling and regulatory networks are shared between different stresses. First, roots are able to sense the stress and activate several physiological adaptive processes. Many abiotic stresses provoke a damage of the membranes and the loss of ionic and osmotic homeostasis that triggers ROS (O_2_^–^, H_2_O_2_) accumulation within different organelles. ROS is produced at the plasma membrane or the mitochondria by NADPH oxidases (RBOHs). This ROS has a dual function in response to abiotic stresses. Although at high levels, ROS are toxic to the cells, they can also act as a signal transducer activating Ca^2+^ channels that causes an increase in the intracellular Ca^2+^ concentration. This flux of Ca^2+^ triggers a cascade of events that activates calcium-dependent protein kinases (MAPKs) that phosphorylate and activate different TFs in the nucleus. These TFs belong to different families including, DREBs, MYBs, NAC, ERFs, WRKY, and HSFs. These TFs can regulate the expression of downstream genes involved in specific gene regulatory networks modulating each abiotic stress. They also regulate the biosynthesis and the metabolism of different phytohormones that in turn coordinate various signal transduction pathways regulating abiotic-stress response. On the other hand, interplay between ROS and hormone signaling orchestrates the acclimation response of plants to different abiotic stress combinations.

Lastly, recent investigations have shown that epigenetic mechanisms play an important role in the regulation of abiotic stress responses. DNA methylation and histone modifications are involved in the response to salinity, drought, and temperature stress (Ashapkin et al., 2020; Miryeganeh, 2021). In rice, the gene *DRM2*, which encodes a DNA demethylase, is upregulated under salinity stress in tolerant cultivars (Ferreira et al., 2015). Cold stress induces the expression of genes encoding histone deacetylases, leading to a global modification of H3 and H4 histones in maize (Hu et al., 2011). In rice, the histone mark H3K4me3 has been related to the response to drought stress (Zong et al., 2020). Another epigenetic mechanism that participates in the response to several abiotic stresses is the regulation by small non-coding RNAs, microRNAs (miRNAs) or small interfering RNAs (siRNAs) (Sunkar et al., 2007). Small RNAs can regulate gene expression by post-transcriptional gene silencing or DNA methylation mechanisms (Waititu et al., 2020). Stresses such as drought, salinity and heat stress induce the expression of many miRNAs in crops like barley, maize and rice, suggesting an active role of these miRNAs in the responses (Wei et al., 2009; Sailaja et al., 2014; Deng et al., 2015). In rice, siRNAs that are involved in oxidation reduction and proteolysis have been associated with the response to drought stress (Jung et al., 2016). In addition, several heat-responsive siRNAs have been identified in *Brassica rapa* (Yu et al., 2013). However, despite the progress made in this field, the involvement of epigenetic mechanisms in abiotic stress tolerance in roots is still poorly known (Figure 1).

## Multi-Stress Combination and Roots

**FIGURE 2:**
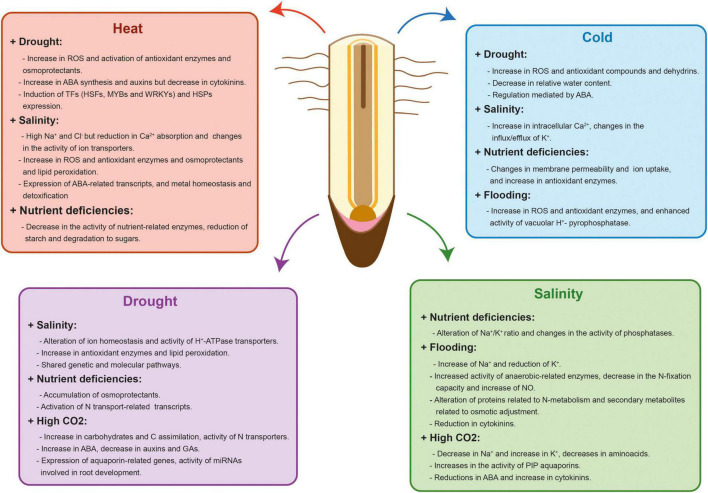
Physiological, biochemical, hormonal and molecular effects of different abiotic stress combinations in plant roots. Red, blue, purple, and green charts show the physiological, biochemical, hormonal and molecular effects that occur in each stress combination. Some of these responses are similar between stresses but plants also trigger specific responses that are tailored to a particular stress combination. In addition, two abiotic stresses that are occurring simultaneously can aggravate or benefit crop survival and growth.

Plants have evolved complex acclimation mechanisms that start with the perception and transmission of the stress signals to the cellular machinery, the subsequent triggering of different signaling pathways and the final activation of an adaptive response. Some of these acclimation responses are similar between stresses but plants can also trigger responses that are tailored to a particular stress combination (Pandey et al., 2015). As a consequence, plant response to stress combinations cannot be easily predicted from studying each single stress individually (Zandalinas et al., 2021). Moreover, two abiotic stresses that are occurring simultaneously can either aggravate or benefit plant survival and growth (Suzuki et al., 2014). Although most of stress combinations have an additive negative interaction (Ahmed et al., 2013b; Alhdad et al., 2013; Sales et al., 2013), there are examples of stresses that interact positively like drought and O_3_ stress or salinity and high CO_2_ (Iyer et al., 2013; Perez-Lopez et al., 2013). Understanding how crop tolerance mechanisms are adjusted to specific combinations of stresses is important to maintain yield stability under the variable environmental conditions driven by climate change. In this section, we will summarize our current knowledge of the main changes affecting RSA and functionality produced by combined abiotic stresses similar to the ones confronted by crops in the field (Table 1 and Figure 2).

**TABLE 1:** Individual effects of each stress and each abiotic stress combination in roots.

Thermal-related stresses		References
*Heat stress*		
Individual effects	- Reduction in root growth - Changes in the fluidity of the cell plasma membrane	- Wu et al., 2020 - Los and Murata, 2004
Combination with drought	- Reduction in root growth - Reduction in the number of lateral roots - Suppression of the development of seminal roots	- Vescio et al., 2021 - Wu et al., 2017 - Fábián et al., 2008
Combination with salinity	- Reduction in root growth - Increase in root/shoot ratio	- Rivero et al., 2014 - Liu F. Y. et al., 2014
Combination with nutrient deficiencies	- Reduction in root growth - Reduction in root length, number of root tips and root surface area - Changes in the distribution of the root system and reductions in diameter	- Tindall et al., 1990 - Luo X. et al., 2012; Luo H. Y. et al., 2012 - Lahti et al., 2005
*Cold stress*		
Individual effects	- Reductions in root growth - Reductions in water uptake	- Richner et al., 1996 - Yadav, 2010
Combination with drought	- Pre-treatment with drought leads to enhanced root system under cold stress. - Pre-treatment with cold leads to an increase in root dry weight, length and increase in number of lateral roots under drought stress - Cold and drought applied at the same time lead to root growth reduction	- Kaur et al., 2016 - Ling et al., 2015 - Hussain et al., 2020
Combination with salinity	- Pre-treatment with cold decreases ion uptake	- Iqbal and Ashraf, 2010
Combination with nutrient deficiencies	- Combination with K deficiency leads to reductions in root length	- Shi-Heng et al., 2021
Combination with flooding	- Changes in the development of adventitious roots	- Ojeda et al., 2004

**Soil-related stresses**		**References**

*Drought stress*		
Individual effects	- Deeper root system under moderate drought - Reductions in root growth under severe drought	- Moles et al., 2018 - Wu et al., 2017
Combination with salinity	- Aggravation of the effects of drought, inhibiting root growth - Increase in root/shoot ratio - Changes in the root tips cell ultrastructure	- Srivastava and Singh, 2009 - Shaheen and Hood-Nowotny, 2005 - Ibrahim et al., 2019
Combination with nutrient deficiencies	- Inhibition of root growth - P-starvation and drought cause modifications on root hairs	- Shi et al., 2017 - Jungk, 2001
*Salinity stress*		
Individual effects	- Reduction in water uptake	- Negräo et al., 2017
Combination with nutrient deficiencies	- P-starvation with salinity causes changes in the development of lateral roots and reductions in root growth	- Kawa et al., 2020 - Abbas et al., 2018
Combination with flooding	- Detrimental effects on the number of adventitious roots, number of lateral roots and root dry weight	- NeSmith et al., 1995
*Nutrient deficiencies*		
Individual effects	- N deficiency causes roots to become longer, with larger cells but reduced solidity - P deficiency causes reductions on root growth - K deficiency causes reductions in root length, area and volume	- Qin et al., 2019 - Mollier and Pellerin, 1999 - Zhang G. et al., 2009; Zhang Z. et al., 2009
*Flooding stress*		
Individual effects	- Reductions in root growth - Expansion of adventitious roots	- Barrett-Lennard, 2003 - Zhang et al., 2017

**Atmospheric-related stresses**		**References**

*High CO_2_*		
Individual effects	- Enhanced root growth - Increase in root mass, length, area and density	- Uddin et al., 2018 - Chaudhuri et al., 1986
Combination with drought	- Enhanced root biomass compared to individual drought stress	- Li Y. et al., 2020
Combination with salinity	- Enhanced root growth when compared to salinity stress alone	- Ratnakumar et al., 2013

*Summary of the current knowledge regarding morphological effects of abiotic stress combinations in roots of crops. The table summarizes individual effects of each abiotic stress in roots as well as the effects of different stress combinations. The different stresses are divided into three categories: thermal-related stresses, soil-related stresses and atmospheric-related stresses. The morphological effects showed in this table have been observed among several crops. References for each statement are shown in the column at the right side of the table.*

### Thermal-Related Stresses

#### Heat and Drought

The continuous increase in the global mean temperatures together with the constant occurrence of drought episodes foresee that heat and drought stresses are the most likely stress combination affecting agriculture in the near future (Lei et al., 2013). The combination of drought and heat stress causes severe yield reductions in many important crops like wheat, maize, rice, soybean, chickpea, and lentils (Dornbos and Mullen, 1991; Prasad et al., 2011; Awasthi et al., 2014; Obata et al., 2015; Sehgal et al., 2017; Lawas et al., 2018a). Water availability is one of the most limiting factors for crop growth. Roots are essential organs in coping with drought stress because they determine the plant access to water and nutrients (Comas et al., 2013). On the other hand, roots are more sensitive to heat stress than the aerial parts of the plant with usually lower optimal growth temperature (Tuteja and Gill, 2013). High temperatures impair the normal functions of roots by affecting the activities of antioxidant enzymes and carbon partitioning and this negative effect is enhanced by lower soil moisture (Barnabas et al., 2008). Moderate drought stress in rice, maize, wheat, soybean, or tomato plants triggers morphological adaptations such as a deeper root system and more root branching to maximize water uptake (Tahere et al., 2000; Nezhadahmadi et al., 2013; Aslam et al., 2015; Battisti and Sentelhas, 2017; Moles et al., 2018). These root adaptations are cultivar-dependent and genetic variation has been observed in crops like wheat, soybean, and tomato (Fenta et al., 2014; Fang et al., 2017; Moles et al., 2018). However, it has been reported that reductions in root growth take place when the drought is more severe (Aslam et al., 2015; Wu et al., 2017). Similarly, reductions in root growth have also been observed during heat stress in wheat, oilseed rape, pepper, and potato (Aloni et al., 1992; Heckathorn et al., 2013; Wu et al., 2017, 2020). But when drought and heat stress are combined, root growth reductions are more pronounced. Maize plants subjected to the combination of both stresses show higher reductions in root growth and lateral root number than under the individual stresses (Vescio et al., 2021). Reductions in the number of lateral roots have also been reported on canola due to the effects of drought and heat stress combination (Wu et al., 2017). In drought-sensitive wheat cultivars, seedlings treated with drought and heat stress show a higher decrease in the development of seminal roots than seedlings subjected to drought stress alone (Fábián et al., 2008). Likewise, tomato plants exposed to the combination of drought and heat stresses display more reduced root growth than the individual stresses (Zhou et al., 2019). Changes in physiological and metabolic processes under heat and drought stress combination have also been reported. Severe drought conditions together with high temperatures provoke a strong inhibition of root respiration rate as well as a reduction in the partitioning of carbon assimilates to the roots (Prasad et al., 2008). Heat and drought stress treatments cause an increase in oxidative stress that is counterbalance by several ROS-detoxification mechanisms, including the activation of antioxidative compounds and enzymes (Zandalinas et al., 2018). Thus, antioxidant enzymes like CAT, SOD, APX, and glutathione reductase (GR) accumulate in roots after exposure to simultaneous drought and heat stresses in maize (Hu et al., 2010). In tobacco roots, APX accumulates under the combination of drought and heat stress and similarly, an increase of SOD, CAT, and GR levels is detected in barley roots (Torun, 2019). Likewise, the level of antioxidant compounds such as malondialdehyde (MDA) increases in citrus roots (Zandalinas et al., 2017). In cotton, proline also accumulates in roots (Sekmen et al., 2014). But in tobacco plants, drought-induced increase in proline and polyamine root levels are not affected further by the simultaneous application of heat stress and drought treatment (Cvikrová et al., 2013). All these results suggest that, although with differential role, increased antioxidant capacity could be a crucial tolerance mechanism of crops to this stress combination.

Another level of regulation of combined stresses is defined by several hormonal crosstalk pathways. ABA is the major hormone playing a role in plant responses to drought stress. When roots sense soil dehydration, ABA is synthesized to maintain root growth and increase root hydraulic conductivity to enhance water uptake. ABA can also act as a long-distance signaling molecule, being transported through the xylem to the shoots to regulate stomatal behavior and leaf expansion to prevent plant dehydration (Zhang J. et al., 2006). ABA has shown to be induced under the stress combination of drought and heat as well as under the individual application (Li et al., 2014). The role of ABA under heat and drought stresses has shown to be related to the regulation of the water status, and the induction of genes that encode for HSPs and oxidative stress (Suzuki et al., 2016; Zandalinas et al., 2016). In maize, proteomic analysis of roots subjected to a combination of drought and heat stresses identified proteins involved in cell growth and division, ion transport, metabolism and signal transduction that are also positively regulated by ABA. These results suggest that ABA may mediate the regulation of these biological processes in response to this stress combination in roots (Liu et al., 2013). Other hormones are also involved in the response to these combined stresses, including cytokinins and auxins (Basu et al., 2016). In tobacco, cytokinin levels increase in response to heat stress, whereas decrease under drought stress. However, in plants exposed to a combination of drought and heat stress, a strong suppression of bioactive cytokinins is observed in both shoots and roots due to an enhanced induction of a cytokinin oxidase/dehydrogenase activity (Dobra et al., 2010). Coincidentally, cytokinin oxidase/dehydrogenase overexpression in tobacco roots alters the antioxidant response of leaves and roots exposed to simultaneous drought and heat stresses affecting plant tolerance (Lubovská et al., 2014). Interestingly, a coincident increase in auxin and cytokinin has been described in response to both stresses in roots. This accumulation of auxins in roots may contribute to the development of the primary root, leading to a deeper root system (Vieten et al., 2007). Auxins can be transported to the roots with the help of ABA and the crosstalk between auxin and ABA is believed to play a major role in the root elongation during abiotic stresses such as heat stress (Xu et al., 2013; Wang et al., 2016). To summarize, a crosstalk between different hormones seems to coordinate different developmental and physiological responses to combined heat and drought in roots.

In wheat, tobacco, and sorghum, the combination of drought and heat stress has shown to activate specifics sets of genes that are very different from the transcriptional profiles under drought and heat stresses applied separately (Rizhsky et al., 2002; Rampino et al., 2012; Johnson et al., 2014). Transcripts that are specifically expressed under the combination of drought and heat stress correspond mainly to HSPs, MYBs, WRKYs, GTP-binding proteins, ROS detoxification proteins, lipid biosynthesis and starch degradation enzymes. All these changes in transcript levels may represent key programs of regulatory gene expression underlying tolerance (Rizhsky et al., 2004). In other species, like poplar, many genes are deregulated in roots by either drought or heat stresses but very few of them are commonly deregulated. Interestingly, these altered genes are involved in the synthesis of hormones like ABA and auxin, or in RNA regulation and transport (Jia et al., 2017). In maize roots, several HSFs regulated by ABA are activated under heat stress as well as the combination of heat and drought stress, but not under drought stress alone (Liu et al., 2013). All these data uncover the complexity of the molecular response of plants to this stress combination and reveal the interest of studying this process to identify putative targets genes to improve crop tolerance under natural environmental conditions.

#### Heat and Salinity

Soil salinity is a challenging environmental problem that affects 20% of the irrigated land worldwide (Qadir et al., 2014; Singh, 2022). Soil salinity reduces productivity not only in crops with high salinity sensitivity as sweet potato, wheat or maize but also in highly tolerant crops as cotton, barley and sugar beet (Zorb et al., 2019). Yield losses associated to soil salinity are expected to worsen in many regions because of the effects of climate change. The increase in global population and food demands will require an expansion of cultivated land. This expansion results in higher irrigation demand that often correlates with poor-quality water, leading to increase soil salinization (Yeo, 1998). Soil salinity can cause two different types of stress to the plant: osmotic stress which prevents the plant uptake of water and nutrients such as K^+^ by the roots; and ionic stress due to the toxic high concentrations of Na^+^ that can cause severe cellular damage (Conde et al., 2011; Negräo et al., 2017). Na^+^ enters the roots passively by non-selective cation channels and actively by transporters from the HKT gene family. Na^+^ can be either pumped-out of the roots or stored into root vacuoles via Na^+^/H^+^ antiporters from the high-affinity potassium NHX family. However, Na^+^ can also be transported to the shoots through the xylem (Munns and Tester, 2008; Maathuis, 2014). High temperatures cause changes in the fluidity of the plasma membrane affecting the transport of some of these ions and modify the expression of ion transporters thus altering ion homeostasis (Los and Murata, 2004; Zhang J. H. et al., 2006).

Several studies have found that the combination of both heat and salinity cause severe reductions in root growth. Tomato plants grown under either salinity, heat or their combination show reductions in root growth. However, these reductions are more pronounced in plants treated with salinity stress alone than in plants treated with either the stress combination or the heat treatment, suggesting that, in this case, heat stress can act as an antagonist of salinity stress (Rivero et al., 2014). This effect has also been observed in barley, where salinity stress causes a larger reduction on root growth than the combination of heat and salinity stresses (Faralli et al., 2015). On the other hand, wheat seedlings treated with the combination of heat and salinity stresses show a greater decrease in root growth than plants treated with salt alone (Hamada and Khulaef, 1995; Keleş and Öncel, 2002). Something similar happened in jatropha, where only plants subjected to this stress combination display a reduction in root growth (Silva et al., 2013). In cherry tomatoes, a reduction in root growth is also observed under combined salinity and heat stress (Liu F. Y. et al., 2014). All these results suggest that the effects of heat and salinity stress combination in roots depend not only on the plant species but also on the crop cultivar.

The combination of heat and salinity stresses have different effects on the plant ion concentrations. These differences may be mediated by alterations in the membrane fluidity that, in turn, affect the activity of membrane ion transporters and their uptake (Fan and Evans, 2015). In jatropha plants, the concentrations of Na^+^ and Cl^–^ are higher in roots of plants subjected to heat and salinity stresses, than under salinity stress alone (Silva et al., 2013). It has been suggested that this accumulation of Na+ in the root could alleviate salinity damage by decreasing its level in leaves and reducing salt-induced photosynthesis alterations. But in citrus, the combination with heat stress aggravates the negative effects of salinity because the higher transpiration rate caused by heat prevents the protective physiological responses to salt stress and increases Cl-intake in leaves (Balfagon et al., 2019). Similarly, reductions on the K^+^ concentration in roots are higher under the salinity stress treatment than under the stress combination (Rivero et al., 2014). Some studies suggest that under heat and salinity stress combination Ca^2+^ channels can also sense perturbations in the plasma membrane caused by stresses leading to a reduction in Ca^2+^ absorption by the roots (Orvar et al., 2000). Accordingly, in cherry tomatoes, water and Ca^2+^ absorption by the roots is reduced under combined salinity and high temperatures (Liu F. Y. et al., 2014). The combination of heat and salinity increases the ROS levels in tomato roots and is subsequently compensated with an increase in the amount of proline to counteract ROS negative effects on growth (Rodrigues et al., 2021). In barley, the expression of the *SOD* and *APX* genes is induced in roots by salinity stress, and this induction last longer under the combination of heat stress and salinity (Faralli et al., 2015). In barley, the *CAT* gene is expressed under salinity stress and under the stress combination in the leaves but not in the roots (Torun, 2019). Other mechanism that plants use to cope with osmotic stress is the accumulation of osmoprotectants which can be amino acids polyols or sugars (Yancey, 2005). Accumulation of osmoprotectants has been observed in response to either salinity or heat stress in several crops (Rasheed et al., 2011; Arif et al., 2020; Nadeem et al., 2020). In jatropha, the concentration of free amino acids on roots is higher under the heat stress treatment but not under the salinity or their combination, and root proline and glycine betaine concentration increases under both treatments. On the other hand, sugars concentration in roots is only increased by heat stress, but not by an excess of salt or the combination of both heat and salinity (Silva et al., 2013). Changes in lipid peroxidation have been observed under the combination of heat and salinity in tomato roots (Rodrigues et al., 2021). Studies regarding gene expression in plants exposed to a combination of heat stress and salinity have mainly focused on shoots rather than roots. In *Arabidopsis*, transcriptomic studies have shown that the transcriptional response to the combination of heat and salinity differs from the response to those stresses applied individually and many of the transcripts involved on the response to this stress combination are related to ABA (Suzuki et al., 2016). In barley roots, the stress combination of heat stress and salinity has shown to activate the expression of some stress-related genes such as *HvDRF1* and *HvMT2* (Faralli et al., 2015). *DRF1* participates in the upregulation of genes involved in ABA response and accumulation in roots, whereas *MT2* is involved in metal homeostasis and detoxification (Go and Kim, 2001; Xue and Loveridge, 2004). Together these results suggest an important role of ABA and metal detoxification signaling pathways in the response to the combination of heat and salinity in roots.

#### Heat and Nutrient Deficiencies

Plants needs to obtain an adequate supply of nutrients to meet the demands of their basic cellular processes. Most of the soils used for agricultural practices worldwide contain limited levels of nutrients or lower nutrient availability for plants (Lynch and St. Clair, 2004). Nutrient uptake and assimilation affects various important processes related with yield such as biomass accumulation, carbon-nitrogen partition, seed and fruit development and seed quality (Pandey et al., 2021). Climate change is having an impact in soil fertility and plant nutrient acquisition and availability, increasing the constraints of crop productivity (St. Clair and Lynch, 2010).

It has been shown that heat stress decreases the amount of nutrients on plant tissues as well as the activity of enzymes involved in nutrient metabolism (Klimenko et al., 2006; Heckathorn et al., 2013; Hungria and Kaschuk, 2014). But temperature effect on nutrient uptake varies depends on the type of nutrient and the crop. Thus, in tomato, warmer soils restrict root growth and nutrient uptake causing a reduction in macro and micro-nutrient levels (Tindall et al., 1990). In Norway spruce, changes in the distribution of the root system and reduction in the root diameter and growth caused by heat stress correlate with less nutrient uptake (Lahti et al., 2005). In other tomato cultivars, as a consequence of poor root growth, heat stress reduces the total N content and assimilation and decreases nitrogen uptake related-proteins in roots (Giri et al., 2017). In two grass species used as fodder for livestock, *Agrostis stolonifera* and *Andropogon gerardii*, high temperature treatment of the roots results in a lower number of roots and an increase in the uptake and partitioning of nitrogen, phosphorous and potassium (DeLucia et al., 1992; Huang and Xu, 2000). In contrast, in maize only a moderate decrease in phosphorus and potassium uptake is detected when they are grown at high temperature (Bravo-F and Uribe, 1981; Bassirirad, 2000).

Regarding the effects of the combination of heat stress and nutrient deficiencies in roots, the information is scarce. Morphological and physiological changes caused by the combination of heat stress and nutrient deficiencies, including reductions in root growth and nutrient accumulation, have been observed. Thus, maize and wheat plants exposed to a combination of heat stress and Mg deficiency show reductions in root biomass and yield as well as lower levels of Mg in roots and shoots compared to control plants (Mengutay et al., 2013). Combination of nutrient deficiency with higher temperatures alters HSP synthesis more than high temperature stress alone (Wang et al., 2014). Lettuce plants subjected to a combination of K deficiency and heat show a greater decrease on root length, number of root tips and root surface area than under the individual stresses. Interestingly, a decrease in the root starch concentration is also detected likely due to the starch degradation into sugars which has shown to play an important role as osmoprotectors (Luo H. Y. et al., 2012; Dong and Beckles, 2019). Most of the arable land is poor in nutrients and temperatures are predicted to keep increasing over the next years. Consequently, in the future, crops are likely to simultaneously encounter heat stress and nutrient deficiency more frequently. To confront this challenge, a better knowledge of the regulation of the plant response to this stress combination is urgently needed.

#### Cold and Drought

Climate change will cause fluctuations in global temperatures and arbitrary weather patterns, including recurrent low temperatures episodes (Gu et al., 2008). Cold stress is one of the main abiotic stress factors that cause important damages to agriculture production (Mboup et al., 2012). Cold stress can be divided into chilling (0 – 20°C) and freezing (<0°C) (Ritonga and Chen, 2020). Cold stress alters root length and morphology, limiting the capacity of the roots to take water and nutrients (Cutforth et al., 1986; Richner et al., 1996). In general, the co-occurrence of cold and drought events worsens crop growth and production. Interestingly, the response mechanisms to cold and drought stresses share some similarities. Low temperatures can negatively affect hydraulic conductance, provoking poor root activity and reducing water uptake that produce a chilling-induced water stress similar to drought stress (Janská et al., 2010; Yadav, 2010). Additionally, it has been reported that both stresses cause a decrease in root biomass and an increase in the amount of antioxidant compounds as MDA in maize roots (Öktem et al., 2008). They also provoke an increase in the activity of root antioxidant enzymes in lentil plants (Hussain et al., 2018). In tobacco roots, both stresses produce an accumulation of dehydrins, a multi-family of very hydrophilic proteins (Beck et al., 2007). Interestingly, Norway spruce plants from freezing-tolerant cultivars display a larger root biomass that correlates with increase drought tolerance (Kang et al., 2003). Something similar happens in *Eucaliptus globulus*, where drought-tolerant cultivars that are more tolerant to freezing have also increased root biomass (Shvaleva et al., 2008). Other similar effects of both stresses are showed at the biochemical and molecular level. For example, the transcription factor *WRKY38* is induced in barley roots after exposure to both drought and cold stresses (Marè et al., 2004). In cassava, overexpression of the *AtCBF3* increases cold and drought tolerance and decreases root yield (An et al., 2016). In summary, all these results suggest that some of the physiological and molecular mechanisms controlling root stress tolerance are common to both stresses. Identifying and characterizing these mechanisms might provide a promising biotechnological tool to minimize the negatives effects of both combined stresses in crops.

But although they share common response mechanisms, it is not clear whether the stress combination of cold and drought interacts positively or negatively (Suzuki et al., 2014). Maize plants subjected to drought, chilling or the stress combination show a reduction on root length and biomass, but this reduction is greater in plants treated with either chilling or the stress combination (Hussain et al., 2020). In roots of chickpea plants, the stress combination of cold and drought causes an increase in ROS together with a decrease in the relative water content (RWC) and the amount of polyamines and those responses are more pronounced in the combination than in the single-stress treatments (Nayyar and Chander, 2004). On the other hand, pre-treatment with drought induces chilling-tolerance in a chilling-sensitive variety of maize (Irigoyen et al., 1996). Similar experiments performed in chickpea show that pre-treatment with mild drought improved cold tolerance, enhancing proline content and improving membrane integrity in roots (Kaur et al., 2016). Pre-treatment with drought stress also leads to a lower amount of H_2_O_2_ in roots of tomato seedlings grown under chilling stress (Ghanbari and Sayyari, 2018). In common bean, chilling produces a reduction in the root water uptake, provoking stomata opening and causing subsequent loss of water and wilting. However, exogenous application of ABA to the root system prior to the chilling treatment causes stomata closing and enhances chilling tolerance (Pardossi et al., 2020). Similarly, the closing of the stomata to protect the plant from drought stress has been correlated with chilling tolerance in maize (Aroca et al., 2003). Something similar takes place when plants are pre-treated with cold and then subjected to drought stress. Cold treatment increases root dry weight, length, and number of lateral roots in oilseed rape plants subjected to drought stress. Moreover, cold treatment improves oilseed rape drought tolerance by enhancing antioxidant enzyme activities, increasing osmotic-adjustment, and reducing lipid peroxidation (Ling et al., 2015). These results suggest that cold treatment can be used to ameliorative and protect crops against damage caused by drought stress and conversely the pre-treatment with drought could induce chilling-tolerance. Since the plant responses to cold and drought stress resemble each other, it seems that exposure to one of the stresses could activate the subsequent response to the second stress. However, more experiments in different crops will be necessary to probe the benefit and efficacy of these strategies.

#### Cold and Salinity

Extreme weather events and freeze-thaw cycles and salinity will coexist in certain agricultural areas in the future (Qadir et al., 2014). Several studies seem to indicate that the plant response to salinity and cold stresses might share some similarities. Expression of *JERF1*, an ABA-induced transcription factor, enhances germination and root growth under salinity and cold stresses in tobacco (Wu et al., 2007). *HvPRP*, a gene encoding for a proline-rich protein, is expressed in *Arabidopsis* roots under salinity and cold stress in cotton (Qin et al., 2013). SA also seems to play a role in the response to both salinity and cold stresses in roots (Miura and Tada, 2014). Thus, exogenous application of SA to roots improves chilling tolerance in banana plants (Kang et al., 2003). Similarly, irrigation of maize plants with SA decreases the levels of H_2_O_2_ and superoxide radicals in roots, increasing chilling tolerance (Wang et al., 2012). Cellular signaling by Ca^2+^ might also play a role in the root response to both stresses. Overexpression of the wheat *CIPK14*, a protein kinase that participates in Ca^2+^ signaling, enhances salinity and cold tolerance, as well as root elongation in tobacco plants (Deng et al., 2013).

Scarcely any research has been done regarding root responses to this particular stress combination. Consequently, it is not clear whether the combination of cold and salinity interacts negatively or positively (Mittler, 2006; Suzuki et al., 2014). Salinity causes a plasma membrane depolarization of root cells, altering the efflux and influx of K^+^ (Shabala et al., 2003). Pre-treatment with chilling improves salinity stress in wheat plants, causing an increased K^+^ and Ca^2+^ uptake by the roots and a decreased Na^+^ and Cl^–^ uptake (Iqbal and Ashraf, 2010). Increases in the amount of Ca^2+^ and K^+^ in roots caused by chilling treatment could be attributed to an accumulation of intracellular Ca^2+^ that in turn alters the depolarization of the plasma membrane, leading to changes in the influx and efflux of K^+^. Several studies have shown that an accumulation of Ca^2+^ in the plasma membrane is an important factor contributing to salinity tolerance (Iqbal and Ashraf, 2007; El-Hendawy et al., 2009). These results suggest that increases in intracellular Ca^2+^ caused by pre-treatment with chilling might play a role in subsequent salinity tolerance.

#### Cold and Nutrient Deficiencies

Climate change is predicted to provoke changes in the snow cover, leading to an increased frequency of freeze-thaw cycles in mid to high latitudes (Song et al., 2017). This soil freeze-thaw process could cause modifications in the soil carbon and nitrogen dynamics and provoke that nutrients such as phosphorous could migrate with runoff and soil water (Henry, 2008; Zhao et al., 2021). Freezing can also lead to losses in inorganic nitrogen from the soil due to leaching, which in turn can decrease the N uptake by the plant roots (Joseph and Henry, 2008). Thus, alterations in N acquisition by roots because of low temperatures have been reported in some crops and forest ecosystems. In maple trees, freezing negatively affects the root uptake of N due to root injury, cellular damage and changes in the root osmotic potential (Campbell et al., 2014). Freezing has also negative effects in the N fixation in soybean by reducing root respiration and causing accumulation of N in root nodules and decreasing N partitioning to young shoot tissues (Walsh and Layzell, 1986). Another aspect of the plants response to cold stress depends on the activity of different phytohormones. It has been described that cold decreases cytokinin levels and increases ABA production by the roots and ABA subsequent transport to the shoots (Tantau and Dörffling, 1991). On the other hand, nutrient deficiencies lead to a decrease in cytokinin production by the roots promoting root growth and nutrient uptake in rice (Wang et al., 2020).

Few studies have focused on the root responses to cold stress in combination with nutrient deficiencies. K deficiency together with cold stress cause a reduction of root length and weight in cabbage (Shi-Heng et al., 2021). Stress combination of P deficiency and low temperature affects the membrane permeability in roots of crotalaria plants (Shen and Yan, 2002). Phosphate deficiency together with cold stress enhance the expression of several photosynthetic genes in plant roots as well as an increase in iron uptake (Gao et al., 2022). Low N and P availability together with chilling stress produce an increase in the amount of CAT, SOD, and POD in roots of cucumber seedlings. This increase in the root activities of antioxidant enzymes have also been observed in cucumber seedlings subjected to chilling stress after exogenous application of N, P, or K (Yan et al., 2012). Several studies have focused on the effects of exogenous nutrient application on plant tolerance to low temperatures (Waraich et al., 2012). The exogenous treatment with N and P seems to affect the freezing tolerance of winter wheat, enhancing root growth and increasing the tissue concentrations of N and P (Gusta et al., 1999). Similarly, the use of Zn improves the tolerance to low temperatures in rice (Liu et al., 2021). Finally, exogenous application of K^+^ causes an increase in root dry weight and RWC as well as an increase in the expression of genes encoding for antioxidant enzymes such as CAT, SOD, and GPX in ginseng plants subjected to chilling stress (Devi et al., 2012).

#### Cold and Flooding

Flooding or waterlogging affects around 10% of the agricultural land (Setter and Waters, 2003; Tewari and Mishra, 2018). It is mainly produced by irregular rainfall and poor soil drainage. Depending on the type of crop and the extension of the stress, total yield loss by waterlogging range from 15 to 80% (Patel et al., 2014). Shifts in precipitation and temperatures lead to the possibility of flooding events occurring together with low temperatures episodes (Dempewolf et al., 2014; Matti et al., 2016). Root response to flooding and chilling stresses combination are poorly known. One of the few responses that has been reported is the alteration of the development of adventitious roots by the combination of flooding and chilling stress on *Annona glabra* and *Annona muricata* (Ojeda et al., 2004). Hypoxia produced by flood increases the redox potential between waterlogged soil and plants, which leads to the production of ROS. Increased expression of *APX* in roots seems to contribute to waterlogging tolerance in tomato (Lin et al., 2004). Similarly, the expression in roots of antioxidant enzymes such as *APX* and *SOD* are related to the tolerance to the combined chilling and waterlogging stresses in winter squash and sponge gourd (Chiang et al., 2014). Root vacuolar H^+^ phosphatases are ion pumps that are present on the vacuolar membrane. They have shown to play a role in the tolerance to abiotic stresses by helping the sequestration of ions in the vacuole (Lv et al., 2015). This enzyme is induced in rice under anoxia stress (Liu et al., 2010). Interestingly, a vacuolar H^+^ – pyrophosphatase is induced under chilling stress combined with anoxia in the roots of rice seedlings, indicating its possible role in the tolerance to this stress combination (Carystinos et al., 1995). More studies on combined flooding and low temperatures in crops are needed to unravel the role of roots in this process.

### Soil-Related Stresses

#### Salinity and Drought

By the year 2025, drought and salinity are expected to affect 50% of the arable land (Wang et al., 2003). Increased salinity can impair the ability of roots to uptake water and produce similar effects to drought (Munns, 2002; Roy et al., 2014). Since salinity disturbs the water uptake by roots, the co-occurrence of drought and salinity in the field can produce serious growth and production problems for crops (Paul et al., 2019). One of the most typical plant adaptations to cope with drought is the allocation of resources to roots in order to enhance its growth and improve the acquisition of water and nutrients (Sharp et al., 2004; Gupta et al., 2020). It has been suggested that salinity could interfere with this response by disturbing root growth and hindering water and nutrients absorption (Srivastava and Singh, 2009). In some cotton cultivars, root biomass declines after exposure to either drought, salinity or the combination of both (Zhang et al., 2013). In poplar cultivars, root biomass decreases more under the stress combination of drought and salinity than under the individual stresses, but root/shoot ratio is maintained in all conditions (Yu et al., 2020). On the other hand, citrus seedlings pre-treated with high level of salt show greater tolerance to drought in later stages of development (Perez-Perez et al., 2007). In wheat, the stress combination of drought and salinity increases the root/shoot ratio as well as the biomass and carbon allocation to roots more than under salinity alone (Shaheen and Hood-Nowotny, 2005). Additionally in this crop, tolerance to the stress combination of drought and salinity seems to be associated with a larger root length in a cultivar-dependent manner (Dugasa et al., 2019). Ion homeostasis could play a role in the tolerance to combined salinity and drought stresses in roots. Thus, accumulation of Na^+^ and Cl^–^ in the root system is one of the responses observed in several crops after exposure to the combination of drought and salinity. Barley plants subjected to salinity alone or the combination of both stresses, drought and salinity, accumulate higher levels of Na^+^ and Cl^–^ while reducing the amount of K^+^ in roots (Ahmed et al., 2013a). In maize, the accumulation of Na^+^ in roots is greater under salinity than under the combination of drought and salinity (Morari et al., 2015). Salt-tolerant cotton cultivars accumulate higher amounts of Ca^2+^ and Mg^2+^ in roots after exposure to the combination of drought and salinity than sensitive cotton cultivars. This increase is accompanied by an increase in the number of vacuoles indicating a possible accumulation of ions in these organelles to avoid their excessive levels in the cytosol (Ibrahim et al., 2019). Similarly, tolerant cultivars treated with the combination of drought and salinity retain more K^+^ on roots than the sensitive ones indicating that accumulation of K^+^ in roots might also play a role in the tolerance to theses combined stresses (Dugasa et al., 2019). It has been showed that the biochemical effects of the combination of drought and salinity include the alteration of antioxidant enzymes levels in roots. In cotton roots after exposure to the combination of drought and salinity, H_2_O_2_ content and O_2_^–^ generation increase whereas antioxidant enzymes activity decreases, leading to lipid peroxidation and the modification of the plasma membrane composition. Moreover, the activity of membrane H^+^-ATPase and Ca2+-ATPase transporters is also more reduced under the stress combination than under each individual stress (Zhang et al., 2013).

Limited information is available regarding the hormonal and molecular effects of the stress combination of drought and salinity. In citrus plants, drought increases ABA synthesis in the roots that is transported to the shoots and produce stomatal closure and leaf abscission (Syvertsen and Garcia-Sanchez, 2014). This drought-induced effect might interact with the plant salinity response since pre-treatment with ABA has shown to increase salt-tolerance in citrus (Gómez-Cadenas et al., 1998). ABA treatment also seems to alleviate salinity stress in other crops such as wheat or tomato (Afzal et al., 2006; Martinez-Andujar et al., 2021). In cotton and wheat, the overexpression of the rice gene *NAC1* enhances drought and salt tolerance by enhancing root development (Saad et al., 2013; Liu G. et al., 2014). Similarly, finger millet NAC67 overexpression increases root growth and also confers tolerance in rice (Rahman et al., 2016). Expression of several genes belonging to the *AREB* and *DREB* families is increased after exposure to drought and salt individually in grapevine roots (Zandkarimi et al., 2015). Transcriptomic analyses in soybean roots show a significant overlapping between drought-stressed and salt-stressed plants suggesting common gene regulatory networks (Fan et al., 2013). In chickpea roots, transcripts that respond to drought and salinity are associated to carbohydrate metabolism, lipid metabolism, redox homeostasis and cell-wall component biogenesis suggesting the participation of these processes in the plant adaptation to these combined stresses (Garg et al., 2016). Although these studies suggest that the root response to drought and salinity stresses might share similarities, more research is needed to understand the regulatory networks underlying these combined stresses.

#### Salinity and Nutrient Deficiencies

Salinity stress is a major thread to agriculture worldwide, particularly in arid or semi-arid lands (Gupta and Huang, 2014). Excess of salt in the soil decreases the capacity of the plant to absorb nutrients linking both stresses, salinity, and nutrient deficiency (Acosta-Motos et al., 2017). Consequently, the combination of both stresses will create an exceedingly adverse environment for plant growth and crop yield. As described before, roots play an important role in the plant response to salinity and nutrient deficiencies. An enlarged and expanded root system with better capability to absorb nutrients from the soil has been associated with the tolerance to osmotic stress in several species such as maize and coffee (Ober and Sharp, 2003; Pinheiro et al., 2005; Sharp et al., 2006). The capacity of roots to absorb and retain K^+^ together with the whole plant K+/Na+ ratio is one of the mechanisms that has been correlated with salt-tolerance (Kumari et al., 2021; Tao et al., 2021). It is already known that soil salinity can induce nutrient deficiency due to the similar chemical properties of Na^+^ and K^+^ (Hasanuzzaman et al., 2018). Under high salinity, Na^+^ can cause the disruption of the activity of root K^+^ channels and low affinity K^+^ channels by acting as Na^+^ transporters (Chinnusamy et al., 2005). Thus, a correlation between roots K^+^ status and salinity tolerance has been described in barley and wheat (Chen et al., 2007; Cuin and Shabala, 2007). On the contrary, in wild barley, *Hordeum maritimum*, root growth is equally affected by salinity under either high or low K^+^ (Hafsi et al., 2007). But in maize, salinity alone has shown to decrease K^+^ uptake as well as K^+^ transport from roots to shoots and this effect is worsened under K^+^ deficiency (Botella et al., 1997). In red beet, low levels of K^+^ in the soil increase the Na^+^ uptake under salinity stress (Subbarao et al., 2000). Additionally, salinity stress in combination with K^+^ deficiency increases the levels of the antioxidant compound MDA in roots of the herbaceous plant *Aeluropus lagopoides* (Alikhani et al., 2011).

Other nutrient deficiencies that might interact with the root responses to salinity stress are P and N deficiencies. In *Arabidopsis*, P-starvation influences root responses to salinity affecting mainly the development of the lateral roots (Kawa et al., 2020). Wheat plants subjected to either low P, salinity or the combination of both show reductions in root growth but the effects of low P and the stress combination increase compare to salinity alone. Furthermore, wheat cultivars differing in salt tolerance are similarly affected by either low P or the stress combination (Abbas et al., 2018). On the other hand, P deficiency together with salinity stress decrease the accumulation of P in maize while salinity stress alone tends to increase the root P concentration in soybean (Phang et al., 2009). The detrimental effects of P deficiency and salinity on plant growth are more pronounced in cultivated barley than in wild barley. This lower effect correlates with a larger root system, higher root/shoot ratio and higher root P content under these stresses (Talbi Zribi et al., 2011; Zribi et al., 2014). Moderate salinity seems to alleviate the P-starvation response in barley by increasing root growth and K^+^/Na^+^ ratio (Zribi et al., 2012). Conversely, P-starvation increases salinity tolerance by increasing the root/shoot ratio, root length, K^+^/Na^+^ ratio and antioxidant capacity in barley (Talbi Zribi et al., 2011). However, another study in barley has shown that exogenous application of P enhances salinity tolerance due to root ion accumulation and root growth induction together with an increase in the levels of proline and soluble sugars in the shoots (Khosh Kholgh Sima et al., 2012). Additionally, the activity of root pyrophosphatases involved in the recycling of free Pi has been involved in the root response to the combination of salinity and nutrient deficiencies. Overexpression of a H^+^-pyrophosphatase from *Salicornia europaea* confers tolerance to the combination of salinity and N deficiency by increasing the amount of sugars and the transport of photosynthates to roots in wheat (Lv et al., 2015). Similar results are found in tobacco, where overexpression of a wheat vacuolar H^+^-pyrophosphatase enhances tolerance to salinity and P and N deficiencies by enlarging the root system and improving nutrient absorption (Li et al., 2014). In maize plants overexpressing a H^+^-pyrophosphatase, the enlargement of the root system is associated to the upregulation of genes related to auxin transport, suggesting a role of this hormone in the response to this stress combination (Pei et al., 2012). Finally, salinity stress might cause changes in the membrane permeability impeding the uptake of heavy metals. But when salinity is combined with N deficiency this effect seems to be blocked favoring the entrance of heavy metals in the root (Cheng et al., 2012). In summary, interplay between ions homeostasis and root development seems to underlie the tolerance to salinity and nutrient deficiency, revealing root development and function-related traits as promising targets to cope with these combined stresses in crops.

#### Salinity and Flooding

Another risk for agriculture associated to climate change is related to the increase in extreme rainfall that is raising the frequency of flooding events. Floods aggravate food insecurity by destroying cropping areas and delaying crop planting due to the associated high soil moisture (Lei et al., 2013; Iizumi and Ramankutty, 2015). Flooding causes changes in soil structure, depletes O_2_, enhances the amount of CO_2_, and increases the amount of elements such as Mg or Fe in the soil (Kozlowski, 1997). The depletion of O_2_ in the soil leads to a switch from aerobic to anaerobic metabolism in roots, accelerating root senescence and reducing root and shoot growth (Barrett-Lennard, 2003; Teakle et al., 2006). Flooding can also cause down-regulation of aquaporins expression, thus reducing root hydraulic conductance (Arbona et al., 2009; Gimeno et al., 2012; Rodríguez-Gamir et al., 2012). An increase in the flooding of coastal regions due to the rising of the sea-level and the alteration of climatology has been predicted for the near future. More frequent waves and storm surges are increasing seawater flooding of coastal regions (Vitousek et al., 2017). As a result, anionic and osmotic stress caused by the high salinity of seawater will become an additional problem besides the low O_2_ and CO_2_ levels caused by anoxia. The most common developmental root response to flooding is the development of adventitious roots and aerenchyma tissue formation. The expansion of the adventitious roots increases plant biomass and due to its high porosity improves water and nutrients uptake under the submerged state (Zhang et al., 2017). On the other hand, aerenchyma tissues enable the passage of oxygen from shoots to roots assisting the oxygenation of submerged tissues (Colmer, 2003).

Salinity stress in combination with waterlogging causes more damage to crops, including their roots, than salinity stress alone (Barrett-Lennard and Shabala, 2013). In summer squash, the combination of salinity and flooding affects the number and length of adventitious roots, number of lateral roots and root dry weight (NeSmith et al., 1995). Additionally, waterlogging can diminish the adaptation strategies that plants use to cope with salinity, such as the exclusion of Na^+^ and Cl^–^ (Kahlown and Azam, 2002). An increase in the level of CO_2_ as a result of flooding is accompanied by phytotoxin accumulation, which inhibits root respiration in soybean (Tewari and Arora, 2016). The absence of O_2_ in the root-zone as a consequence of flooding events also causes an increase in the uptake of Na^+^ and a decrease in K^+^ in roots of several species such as wheat, barley, and cotton (Barrett-Lennard et al., 1999; Pang et al., 2006; Ashraf et al., 2011). Consequently, in barley, a greater increase in root Na^+^ and a decrease in K^+^ is produced under combined salinity and flooding than under salinity stress alone (Zeng et al., 2013). As it has been mentioned, the lack of O_2_ in the soil leads to a switch from aerobic respiration to anaerobic respiration reducing the production of ATP and the activity of root H^+^-ATPases that participate in Na^+^ and K^+^ homeostasis (Zeng et al., 2013). In wheat, it has been showed that salinity intensifies the negative effect of waterlogging at all the growth stages and affects root growth (Saqib et al., 2013).

In addition, hypoxia in combination with salinity stress increases the activities of two enzymes related to anaerobic fermentation, the alcohol dehydrogenase (ADH) and the lactate dehydrogenase (LDH), and reduces aerenchyma formation in roots of wheat cultivars (Akhtar et al., 1998). In alfalfa, the stress combination of flooding and salinity also decreases the root nitrate fixation capacity but increases the production of NO that participates in the formation of lysigenous aerenchyma to enhance O_2_ diffusion through the roots (Wany et al., 2017; Aridhi et al., 2020). In Arabidopsis roots, the NADPH/respiratory burst oxidase protein D (RBOHD) enzyme mediates H_2_O_2_ formation and Ca^2+^ signaling under salinity and waterlogging, affecting ion homeostasis and reducing Na^+^ accumulation (Wang et al., 2019). Additionally, the combination of salinity and waterlogging seems to reduce the cytokinin levels in sunflower (Burrows and Carr, 1969). In tomato and rice, expansion of adventitious roots under flooding is regulated by ET as well as root growth under salinity, suggesting a common pathway of ET-mediated regulation of RSA by both stresses (Sauter, 2013; Tao et al., 2015). Finally, proteomic analysis in soybean seedlings has shown that proteins responding to salinity and waterlogging stress combination are mainly involved in the maintenance of energy and N metabolism, osmotic adjustment-related secondary metabolites such as anthocyanins and flavonoids, and protein trafficking and signaling (Alam et al., 2011). Further characterization of this response, specially focused on roots, will shed light on the main processes involved in the tolerance to salinity and flooding.

#### Nutrient Deficiencies and Drought

Besides the detrimental effects that drought has in plant growth and development, this stress seems to alter mineral nutrition. Nutrient acquisition by roots is highly dependent on soil moisture and nutrient transport from roots to shoots relies mainly on leaf transpiration which is also highly influenced by the water status of the soil (da Silva et al., 2011). Water deficiency leads to the development of deeper root system to absorb water from deeper layers of the soil. This effect might be detrimental under nutrient deficiencies where a shallow and superficial root system needs to be developed to acquire some of the main nutrients that are typically distributed in the superficial areas of the soil (Ho et al., 2005). In wheat cultivars, heterogeneous distribution of nutrients in the soil enhances root growth in the areas where the nutrients are more abundant. Remarkably, the root sections growing in the nutrient-rich zone synthesize higher levels of ABA which has been linked to deeper roots and drought tolerance (Trapeznikov et al., 2003). In catalpa, N deficiency together with drought stress inhibits root growth. Conversely, exogenous application of N greatly reduces the effects of drought on roots by upregulating the expression of genes related to ABA biosynthesis and enhancing ABA signaling as well as the crosstalk with JA and auxin. Moreover, under severe drought stress, N uptake is impaired in maize roots. However, when a moderate drought stress is applied, no reduction in the capacity to acquire N is observed (Buljovcic and Engels, 2001). Adequate supply of N also enhances drought tolerance in poplars, improving the water uptake by the root system (Lu et al., 2019). In maize, the tolerance to the stress combination of N deficiency and drought seems to be cultivar-dependent and correlates with genotypes with enlarged root system (Eghball and Maranville, 1993). Interestingly, drought-tolerant sugar beet cultivars produce more root biomass as well as more root glycine betaine under combined drought and nitrogen deficiency, linking robust roots systems with their tolerance to the combination of both stresses (Shaw et al., 2002). Transcriptome analysis in poplar roots identify several transcripts that are specifically regulated under the combination of low N and drought. Most of the transcripts related to ammonium transporters and uptake are increased suggesting a preference for ammonium as a nitrogen source under these combined stresses (Zhang et al., 2018). Modulation of root length and RSA have shown to play an important role in drought tolerance and P-starvation in common bean (Margaret et al., 2014). Additionally, root hairs are essential for nutrient uptake, especially on P acquisition (Jungk, 2001). Barley genotypes with less root hairs are much more sensitive to the stress combination than wild type plants (Brown et al., 2012). Root hairs also play an important role on tolerance to drought and P-starvation in maize (Klamer et al., 2019). Drought-tolerant maize cultivars develop larger root system, root surface area and volume under P-starvation than drought-sensitive cultivars (Cantão et al., 2008). And in groundnut, the presence of larger roots, higher root density and root dry matter correlates with the tolerance to combined drought and P-starvation indicating the importance of these root traits in the plant adaptation to these stresses (Falalou, 2018).

### Environmental and Atmospheric-Related Stresses

#### High CO_2_ and Drought

The levels of atmospheric CO_2_ have been continually raising since the industrial revolution and will continue to increase in the future (Ciais et al., 2013). Apart from its indirect effects on the environment, elevated CO_2_ levels have direct effects in crops mainly affecting the photosynthesis rate (Dusenge et al., 2019). Elevated CO_2_ not only affects the aerial parts of the plant but also the root system. High CO_2_ levels increase root length and diameter and modify root nutrient acquisition (Nie et al., 2013). Drought stress triggers stomatal closure to avoid water loss but also decreases CO_2_ absorption diminishing photosynthesis and negatively affecting plant growth (Li S. et al., 2020). The effects of elevated atmospheric CO_2_ in combination with drought stress cause a positive interaction and might mitigate each other’s negative effects (Mittler, 2006; Suzuki et al., 2014). Soybean plants subjected to drought and high CO_2_ allocated the same biomass to the roots than plants exposed to drought stress alone (Li et al., 2013). Additionally, the increase in photosynthesis and water use efficiency produced by elevated CO_2_ could improve the amount of C assimilation and carbohydrates synthesis, having a positive effect on root growth (Sun et al., 2012; Yan et al., 2017; Uddin et al., 2018). Thus, elevated CO_2_ has been correlated with enhanced root biomass, length, area and density due to an increase in carbon assimilation in sorghum (Chaudhuri et al., 1986). An increase in root length, as a consequence of elevated CO_2_ and drought, has been observed in several crops such as wheat, barley and coffee (Chaudhuri et al., 1990; De Souza et al., 2015; Avila et al., 2020; Bista et al., 2020). In addition to the effect on root growth, elevated CO_2_ also enhances root respiration in drought-stressed pepper plants (del Amor et al., 2010). In cucumber, elevated CO_2_ increases root biomass and hydraulic conductivity under moderate drought stress, and regulates the expression of aquaporin-related genes (Li Y. et al., 2020). In barley, the exudation of sugars and carbon is lower in elevated CO_2_ conditions than in ambient CO_2_ under well-watered regimes. However, if plants are exposed to drought stress, the exudation of sugars and carbon is higher under elevated CO_2_ (Zandkarimi et al., 2015). Elevated atmospheric CO_2_ produces changes in nutrient uptake under drought stress. In barley, drought stress alone significantly reduces the uptake of N and P but the increase of CO_2_ has no clear effect. However, high levels of CO_2_ and drought cause a mild increase in the activity of NRT1 and AMT1 which are root transporters for NO_3_^–^ and NH_4_^+^, respectively (Bista et al., 2020). This observation could indicate a positive effect in the uptake of nutrients under drought stress caused by elevated CO_2_. Finally, epigenetic regulation has shown to play a role in the root responses to the combination of drought and elevated atmospheric CO_2_. Several drought–induced miRNAs that participate in carbon fixation, starch and sucrose metabolism, and hormone regulation have shown to respond to this stress combination in sweet potato storage- roots (Saminathan et al., 2019).

#### High CO_2_ and Salinity

Elevated atmospheric CO_2_ is likely to interact with salinity in some areas of the agricultural land. Although the combined effects of high CO_2_ and salinity stress on crops are not well understood, few studies have suggested that they might have antagonistic effects. Thus, high CO_2_ might mitigate the negative effects of salinity stress on plants. In peanut, root growth under salinity stress is enhanced by elevated CO_2_ (Ratnakumar et al., 2013). In sorghum, elevated CO_2_ has beneficial effects in root biomass under saline conditions. High CO_2_ levels also decrease Na^+^ and increased K^+^ on salt-stressed roots compared to plants treated with salinity and ambient CO_2_ suggesting a positive effect of CO_2_ in ion homeostasis of salt-stressed roots (Keramat et al., 2020). In olive trees, elevated CO_2_ decreases the levels of Na^+^ and Cl^–^ in roots of salt-sensitive cultivars but has no effect on ion concentrations of salt-tolerant cultivars roots (Melgar et al., 2008). In citrus cultivars, the decrease in the levels of Na^+^ in salt-stressed roots as a consequence of elevated CO_2_ is also observed (García-Sánchez and Syvertsen, 2006). On the contrary, the levels of Na^+^ on salt-stressed roots are not affected by high CO_2_ in tomato (Takagi et al., 2009). In broccoli, enhanced CO_2_ improves the tolerance to salinity by enhancing the activity of root PIP aquaporins that modulates water balance (Zaghdoud et al., 2013). In tomato, elevated CO_2_ causes a greater tolerance to salinity stress due to the reduction of ABA and the ET precursor (ACC) levels and enhanced root growth (Brito et al., 2020). In pepper, elevated CO_2_ also causes a reduction on the amount of ABA and an increase in cytokinin levels in roots under salinity stress when compared to salinity and ambient CO_2_. This increase in the level of cytokinin could prevent the downregulation of the photosynthesis (Piñero et al., 2014). In summary, although high CO_2_ seems to mitigate the negative effects of salinity stress more experiments are needed to confirm this conclusion.

## Conclusions and Future Perspectives

**FIGURE 3:**
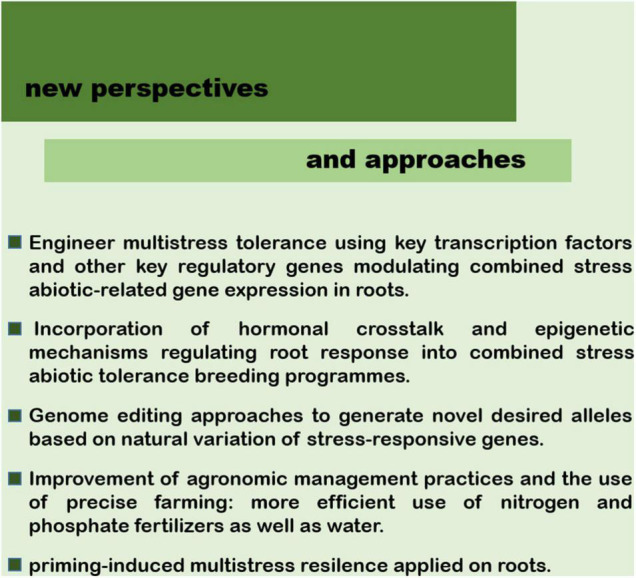
New perspectives and approaches to improve crop root adaptation to multiples stress driven by climate change. Climate change is threatening agricultural productivity. High temperatures together with an increase in atmospheric CO_2_ are leading to changes in the rainfall patterns and increase frequency of extreme weather events like drought, freezing and heat waves. The combination of these environmental changes severely reduce crop yield. To be able to cope with the negative effects of climate change and to guarantee food security is crucial to develop crops that are more resilient to these new combined environmental conditions. In this context, robust and better adapted root systems withhold the potential to reach this goal. Moreover, new perspectives and approaches in the implementation of the knowledge of the role of root traits in the adaptation of crops to combined abiotic stresses will be needed to face this challenge.

Roots are essential to detect and respond to many of the abiotic stresses caused by climate change. For this reason, root adaptive traits constitute an attractive target for future breeding programs trying to address cross-tolerance to multiple abiotic stresses related to climate change. As we have summarized in this review, plant research has only recently started to focus on abiotic stress combinations and the role of roots in these abiotic stress responses is still not well understood. To achieve multi-stress tolerance in roots, first, we need to identify the stress signaling and regulatory pathways shared across stresses. Several key TFs and other regulatory factors modulating combined abiotic stress-related gene expression has already been identified and could be potential genetic tools for cross-tolerance (Yoon et al., 2020). Similarly, recent advances in the common role of hormonal crosstalk and epigenetic mechanisms between stresses could also provide new targets for improving crop resilience. Once major genetic targets are identified and characterized, we will need to reliable and sustainable engineer multi-stress tolerance into crops. In this context, genome editing and cisgenesis are being proven to be a straightforward strategy to introduce desirable root traits and genes into different species of crops (Li et al., 2018). Another interesting strategy to use beneficial root traits to enhance multi-stress tolerance is to take advantage of plant natural variation. Wild relatives from important commercial crops that are tolerant to many abiotic stresses constitute an important source of genetic resources that can be used in breeding programs to achieve tolerance to abiotic stresses in commercial cultivars (Nevo and Chen, 2010; Atwell et al., 2014). Another emerging approach with great potential in cross stress tolerance is based on the advances in our understanding of plant stress memory and priming. Induced short or long-term memory enable crops to be tolerant to additional stresses after exposure to a primary stress or priming agent (Choudhary et al., 2021; Srivastava et al., 2021). The next challenge will be to find the way to incorporate concurrent stresses to this type of strategies (Kollist et al., 2019). Climate change is also enhancing the expansion and virulence of crop root-pathogens (Singh et al., 2018; Savary et al., 2019). Combination of abiotic stresses with biotic stresses in the field increase yield losses even further (Daami-Remadi et al., 2009; Suzuki et al., 2014; Anunda et al., 2019). A better understanding of the crosstalk between environmental conditions and pathogen interaction with plants will be required to produce disease-resistant crops also resilient to climate change. In summary, combined abiotic stress tolerance is a fast-developing field due to the urgency of finding solutions to confront climate change impact on crop productivity. In this context, more efforts on discovering new insights and approaches to understand the role of root systems in abiotic stress will be needed to incorporate beneficial root traits as a valuable tool to enhance combined abiotic stress tolerance (Figure 3).

## Author Contributions

MS-B, JCP, and MP drew the figures, wrote and revised the manuscript. All authors contributed to the article and approved the submitted version.

## Conflict of Interest

The authors declare that the research was conducted in the absence of any commercial or financial relationships that could be construed as a potential conflict of interest.

## Publisher’s Note

All claims expressed in this article are solely those of the authors and do not necessarily represent those of their affiliated organizations, or those of the publisher, the editors and the reviewers. Any product that may be evaluated in this article, or claim that may be made by its manufacturer, is not guaranteed or endorsed by the publisher.
